# MedDiet adherence score for the association between inflammatory markers and cognitive performance in the elderly: a study of the NHANES 2011–2014

**DOI:** 10.1186/s12877-022-03140-1

**Published:** 2022-06-21

**Authors:** Shuting Liu, Xiaorong Chen

**Affiliations:** 1 Internal Medicine Department of the Fifth Outpatient Department, Jinling Hospital Affiliated to Medical School of Nanjing University, Nanjing, 210002 Jiangsu PR China; 2 Neurologic Center, Suining Central Hospital, No.127 Desheng West Road, Chuanshan District, Suining, 629000 Sichuan PR China

**Keywords:** Cognition, MedDiet adherence score, Inflammation, Older adults, NHANES

## Abstract

**Background:**

To explore the optimal Mediterranean diet (MedDiet) adherence score threshold for the association between inflammatory markers and cognitive performance among older adults.

**Methods:**

In this cross-sectional study, we selected data of the elderly (≥ 60 years old) from the National Health and Nutrition Examination Survey (NHANES) 2011–2014 (*n* = 2830). Participants completed at least one cognitive measurement and two 24-h food recalls. By analyzing the relation between inflammatory markers and cognitive performance using the subdivided MedDiet adherence score, we got the optimal MedDiet adherence score threshold. Then the optimal threshold was used to divide participants into high and low MedDiet adherence groups and multivariate logistic regression models were developed to examine the association between inflammatory markers and cognitive performance in each group. Subgroup analysis was conducted based on gender, race, BMI, physical activity level, and chronic diseases.

**Results:**

We chose 4 as the optimal MedDiet adherence score threshold and included these participants whose MedDiet adherence score was 4 or above into the high MedDiet adherence group, while the MedDiet adherence score of the low adherence group was less than 4. We found that the increased white blood cell (WBC) count (OR = 1.44, 95% CI: 1.09–1.90, *P* = 0.008), neutrophil count (OR = 1.30, 95% CI: 1.03–1.65, *P* = 0.023), and neutrophil-albumin ratio (NAR) (OR = 1.34, 95% CI: 1.06–1.70, *P* = 0.012) were all related to a higher risk of low cognitive performance in the low MedDiet adherence group. A higher PLR was linked to a reduced risk of low cognitive performance (OR = 0.86, 95% CI: 0.74–1.00, *P* = 0.036) in the high MedDiet adherence group. Significant differences were found in the associations of WBC count, neutrophil count and NAR with low cognitive performance between the low and high MedDiet adherence groups (all *P* < 0.001). The weakened negative association between inflammatory markers and cognitive performance in the high MedDiet adherence group also existed among male, non-Hispanic white, normal-weight, overweight, moderate work activity, moderate recreational activity, non-depression, hypertension, non-hypertension, non-diabetes, non-stroke, non-heart failure, non-coronary heart disease, or non-heart attack subpopulations of older adults.

**Conclusions:**

The optimal threshold for the MedDiet adherence score was 4, and the negative association between inflammation and cognitive performance could be weakened in older adults whose MedDiet adherence score was ≥ 4.

**Supplementary Information:**

The online version contains supplementary material available at 10.1186/s12877-022-03140-1.

## Background

As the aging population is steadily on the rise, the number of people beyond the age of 60 is estimated to reach 2.1 billion by 2050 [1]. For individuals, aging leads to a decline in the functions of most organs and tissues, and brain aging may give rise to different degrees of behavioral dysfunction and cognitive decline [2], which would cause decreased quality of life [3]. Many studies in the aging field have found that diet and nutrition are significant modifiable factors that may be beneficial to slow down the rate of age-related declines in cognition [4, 5]. The Mediterranean diet (MedDiet) represents a traditional dietary pattern followed by the populations residing in Italy, Spain, Greece, and other countries bordering the Mediterranean basin [6]. Inflammation was identified to be associated with cognitive decline in the elderly [7, 8]. The MedDiet becomes a promising approach in the prevention of cognitive decline due to the direct and/or indirect impact of its anti-inflammatory nutritional constituents on cognitive decline [9–11].

Even though higher MedDiet adherence is good for protecting or maintaining cognitive health, lacking the minimum for reaching high MedDiet adherence puts a huge burden on researching into the relationship between MedDiet adherence and cognitive decline as well as further promoting the MedDiet. Owing to the lack of clear-cut criteria about high MedDiet adherence, people may eat excess food to realize cognitive health protection, making the MedDiet hard to promote in needy families and areas where enough foods about the MedDiet is difficult to obtain [12, 13]. Moreover, the difficulty of changing older adults’ eating habits which have been cultivated for a long time also contributes to the challenges of promoting the MedDiet. Thus, it is a valuable question that how to reach high MedDiet adherence with less effort while protect or maintain cognitive health more efficiently. Most of recent observational studies only reported that high MedDiet adherence benefited the relief of cognitive decline [5, 14–16], and few focuses on how to distinguish between high and low MedDiet adherence.

This cross-sectional study intended to explore the optimal MedDiet adherence score threshold in terms of the association between inflammatory markers and cognitive performance among older adults, using data from the National Health and Nutrition Examination Survey (NHANES) 2011–2014.

## Materials and methods

### Study population

This cross-sectional study utilized data from the NHANES from 2011 to 2014. NHANES data and the variable codebook described are freely available at https://www.cdc.gov/nchs/nhanes/default.aspx. The NHANES used a multistage probability sampling design to produce a weighted, representative sample of the American population [17], and its data sets were collated by the United States National Center for Health Statistics [18]. The National Center for Health Statistics Research Ethics Review Board approved all NHANES protocols, and all participants gave informed consent. The elderly aged 60 years and above who underwent cognitive assessment were included in this study. Those without information on inflammatory markers were excluded.

### Dietary intake assessment

Two 24-h food recalls were conducted by trained dietary interviewers. The first food recall interview was an in-person interview, and interviewers used a standard set of measuring guides to help the respondent report the volume and dimensions of the food items or beverage consumed the day before, from morning till evening. Upon completion of the first interview, measuring tools and guides would be given to these participants. The second food recall interview was a telephone interview and was scheduled 3 to 10 days later. In this interview, tools and guides mentioned above would be used for reporting food amounts [19]. All interview data of each participant were aggregated as average intake from the two days. Then these nutrient and individual food data were quantified and combined into 37 food and beverage groupings called after the Food Patterns Equivalents Database (FPED), which was publicly available [20, 21].

### MedDiet adherence assessment

The total MedDiet score ranged from 0 to 18 points, and higher scores reflected greater MedDiet adherence. The method to calculate the MedDiet adherence score was referred to MedDiet Index [21]. Because alcohol, olive oil, fruits, and vegetables were difficult to know particular categories and quantities, these items are calculated with slight modifications (Table 1). All other MedDiet component scores were calculated following Sofi et al. [21].

**Table 1:** Adherence score of specific MedDiet components

Variables	Intake	MedDiet adherence score
Alcohol	> 24 g/day	0
	< 12 g/day	1
	12-24 g/day	2
Olive oil	< 14 g/day	0
	14–28 g/day	1
	> 28 g/day	2
Fruit	< 1 CE/day	0
	1–2 CEs/day	1
	> 2 CEs/day	2
Vegetables	< 0.5 CEs/day	0
	0.5–1 CE/day	1
	> 1 CE/day	2
Dairy products	> 270 g/day	0
	180–270 g/day	1
	< 180 g/day	2
Legumes	< 70 g/day	0
	70–140 g/day	1
	> 140 g/day	2
Fish	< 100 g/day	0
	100–250 g/day	1
	> 250 g/day	2
Meat and meat products	> 120 g/day	0
	80–120 g/day	1
	< 80 g/day	2
Cereals	< 130 g/day	0
	130–195 g/day	1
	> 195 g/day	2

*MedDiet* Mediterranean diet, *CE* Cup equivalent

### Cognition assessment

Three tests named the Consortium to Establish a Registry for Alzheimer’s Disease (CERAD), the Animal Fluency Test (AFT), and the Digit Symbol Substitution Test (DSST) were applied to assess the cognitive functioning of the participants.

### CERAD

The CERAD test was made up of three consecutive learning trials as well as a delayed recall, which were used to evaluate immediate and delayed learning ability for new verbal information [22]. In the learning trials, after participants learned 10 unrelated new words for a few minutes, they were required to recall these words immediately. The CERAD immediate learning score of each participant was presented as an average number of the total right answers across the three trials. After AFT and DSST finished, the delayed recall would be scored by asking participants to recall 10 words mentioned above again. The sum of the scores for the three immediate trials and the delayed recall trial was the total score of the CERAD.

### AFT

The AFT was commonly used to evaluate categorical verbal fluency that was a vital domain of executive function [22]. The AFT required participants, who had passed the practice test of naming three clothing items before, to answer with as many animal names as possible in one minute. The total score of the AFT was summarized as the number of correct answers.

### DSST

The DSST test is a performance module of the Wechsler Adult Intelligence Scale, which was used to assess processing speed, sustained attention, and working memory among the participants [22, 23]. In the test, participants were given a key grid of numbers and corresponding symbols and a test section with numbers and empty boxes, and were asked to paint as many empty boxes (133 matching boxes in total) as possible with a symbol correctly pairing with each number. The score was the number of correct number-symbol matches achieved within two minutes.

### Calculation of age-dependent cognitive Z-scores

In order to distinguish low cognitive performance from normal cognitive performance, we used the comprehensive score that consisted of the CERAD, AFT, and DSST scores. Because age was a major risk factor for cognitive decline, we calculated age-dependent z-scores for each participant. Individuals were stratified by three age levels: 60–69, 70–79, 80 and above. Age-dependent z-scores for each cognitive test were centered and scaled to have a mean of 0 and a standard deviation of 1 within each age level. A global cognitive measure was calculated as the average of standardized scores from each individual cognitive test. Individual and global standardized cognitive scores <  − 1 were characterized as “low cognitive performance” for their respective cognitive measure.

### Inflammatory markers

Data on white blood cell (WBC) count, neutrophil count, lymphocyte count, neutrophil–lymphocyte ratio (NLR, neutrophil count/lymphocyte count), platelet-lymphocyte ratio (PLR, platelet count/lymphocyte count), and neutrophil-albumin ratio (NAR, neutrophil count/albumin) were obtained as part of the NHANES medical examination. Measures and details can be found in the NHANES Laboratory/Medical Technologists Procedures Manual [24].

### Covariates

Categorical variables in this analysis were gender, race/Hispanic origin, marital status, education, annual family income, sleep disorders, smoking status, alcohol use, work activity, recreational activity, depression, hypertension, diabetes, stroke, heart failure, coronary heart disease, and heart attack. Continuous variables in this analysis were age, body mass index (BMI), sleep time. Specifically, race/Hispanic origin was categorized as Mexican American, Non-Hispanic White, Non-Hispanic Black, and other races. Marital status was categorized as married, widowed/divorced, never married, and living with a partner. Education was categorized as less than high school, high school/general education development (GED), and college or above. Annual family income was categorized as < $20,000 and ≥ $20,000. Sleep disorders were evaluated with the question “Ever told by the doctor have a sleep disorder?” (Yes/No). Smoking status was assessed by the question “Do you smoke cigarettes now?” (Yes/No). Alcohol use was evaluated by the question “In the past 12 months did you have at least 12 drinks of any kind of alcoholic beverage?” (Yes/No). Hypertension, diabetes, stroke, heart failure, coronary heart disease, and heart attack were assessed by the question “Have you ever been told by a doctor that you have…?” (Yes/No). Vigorous work activity was assessed with the question “Does your work involve vigorous-intensity activity that causes large increases in breathing or heart rate like carrying or lifting heavy loads, digging, or construction work for at least 10 min continuously?” (Yes/No). Moderate work activity was evaluated by the question “Does your work involve moderate-intensity activity that causes small increases in breathing or heart rate such as brisk walking or carrying light loads for at least 10 min continuously?” (Yes/No). Depression was assessed by a nine-item depression screening instrument, the Patient Health Questionnaire, and depression was evaluated on the condition that the total score was equal or greater than 10. For subgroup analysis, BMI was converted into a categorical variable, with < 18.5 kg/m^2^ defined as underweight, 18.5–24.9 kg/m^2^ defined as normal-weight, 25.0–29.9 kg/m^2^ defined as overweight, and ≥ 30.0 kg/m^2^ defined as obese.

### Statistical analysis

For the weighted large sample, the measurement data described by mean (standard error) [Mean (SE)] were approximately normally distributed, and the independent-samples weighted t-test was used for the comparison between groups. Enumeration data were expressed as the number of cases and the composition ratio [n (%)], and the comparison between groups was performed using the χ2 test or Fisher’s exact test. The multiple imputation method was adopted to handle with missing data in covariates.

First, we conducted univariate difference analysis to get potential covariates. Then three logistic regression models were established: Model 1 was a univariate model without adjustment; Model 2 and 3 included these covariates in the multivariate logistic regression analysis along with the MedDiet adherence score and inflammatory markers to explore statistical associations of the MedDiet adherence score and inflammatory markers with low cognitive performance. Model 2 was adjusted for age and gender, while Model 3 was adjusted for age, gender, BMI, race/Hispanic origin, marital status, education, annual family income, sleep disorders, alcohol intake, recreational activities, depression, hypertension, diabetes, stroke, and heart failure. Subsequently, we used the subdivided MedDiet adherence score to evaluate the relations between the inflammatory markers and low cognitive performance to get the optimal MedDiet adherence score threshold. Afterwards, the optimal threshold was used to divide participants into high and low MedDiet adherence groups and the three logistic regression models were developed to demonstrate the difference in the association of inflammatory markers and low cognitive performance between the low and high MedDiet adherence groups, so as to assess whether the optimal threshold was appropriate based on whether the negative association of inflammatory markers and low cognitive performance became weak in the high MedDiet adherence population. Further, subgroup analysis was conducted to illustrate whether this difference in the association was consistent across different subpopulations, including different gender, race, BMI, physical activity level, and chronic disease (depression, hypertension, diabetes, stroke, heart failure, coronary heart disease, heart attack) populations.

All analyses were carried out using SAS 9.4 (SAS Institute, Cary, NC), and all data were weighted with WTMEC2YR, SDMVPSU and SDMVSTRA as weighting variables. All statistical tests were two-sided, and statistical significance was set at *P* < 0.05.

## Results

### Characteristics of the included participants

**Fig. 1:**
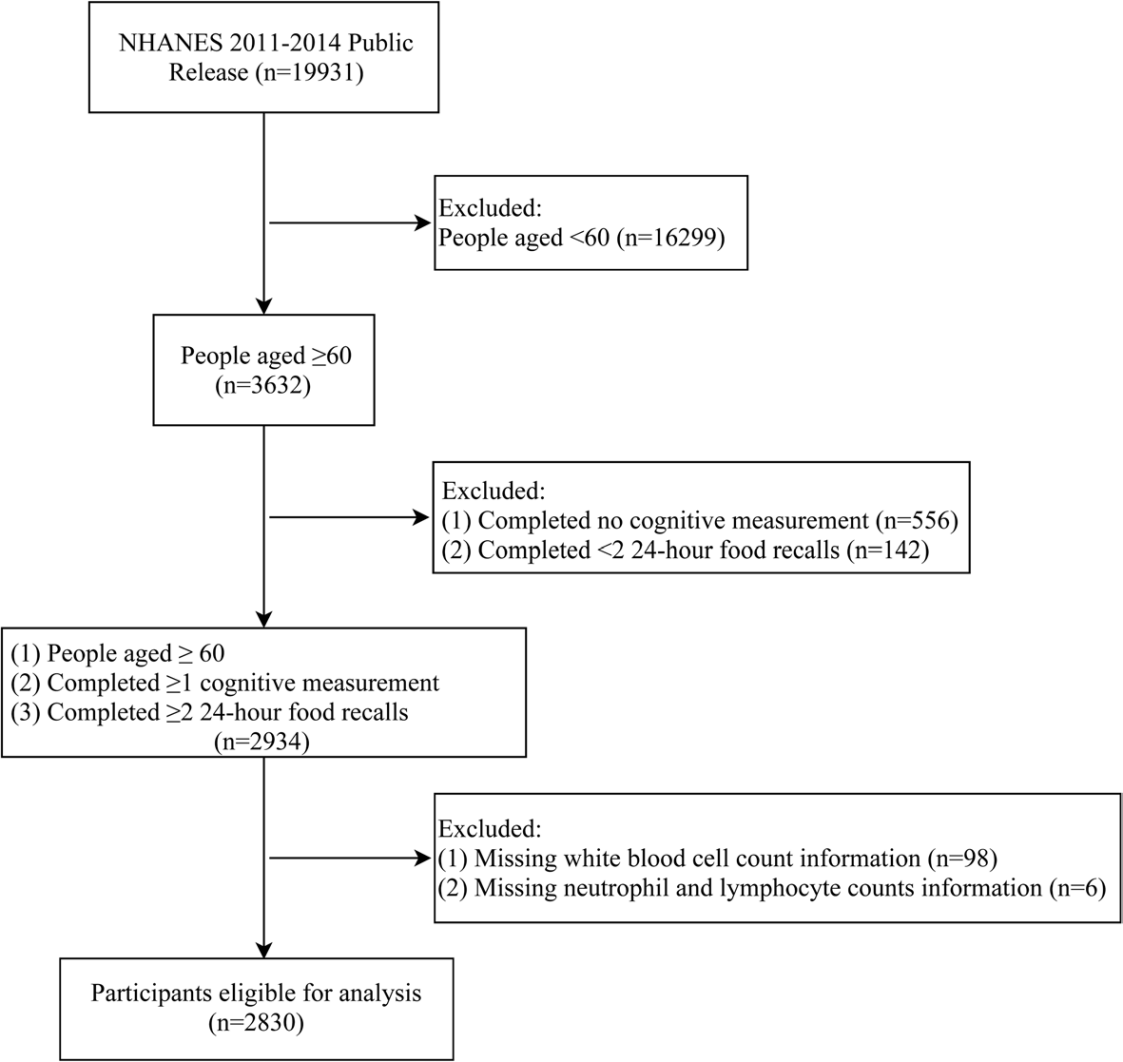
Flowchart for participant selection

A flowchart for participant selection is indicated in Fig. 1. People who (1) were younger than 60 years old (*n* = 16,299), (2) did not complete any cognitive measurement (*n* = 556), (3) did not complete two 24-h food recalls (*n* = 142), and (4) had missing data of inflammatory markers (white blood cell count, neutrophil and lymphocyte counts) (*n* = 104) were excluded. In the end, a total of 2830 participants were included in the study.

Table 2 displays the basic demographic characteristics of the participants. The average age was 69.22 years, and 54.42% were females. The mean BMI was 29.06 kg/m^2^, and the majority (80.04%) of the participants were non-Hispanic whites. Most were married (62.28%), had college education or above (61.66%), and earned money not less than $20,000 per year (82.66%). 27.27% were current alcohol drinkers. Of these subjects, 7.23% had depression, 58.83% had hypertension, 20.68% had diabetes, 6.51% had a stroke, and 6.70% had heart failure.

**Table 2:** Baseline characteristics of the normal and low cognitive performance groups

Variables	Total (*n* = 2830)	Normal cognitive performance group (*n* = 2351)	Low cognitive performance group^a^ (*n* = 479)	*P*
Age, Mean (SE)	69.22 (0.19)	69.04 (0.20)	71.07 (0.47)	< 0.001
Gender, n (%)				0.772
Male	1380 (45.58)	1123 (45.67)	257 (44.61)	
Female	1450 (54.42)	1228 (54.33)	222 (55.39)	
BMI, Mean (SE)	29.06 (0.24)	29.02 (0.25)	29.46 (0.46)	0.371
Race/Hispanic origin, n (%)				< 0.001
Mexican American	249 (3.36)	175 (2.58)	74 (11.67)	
Non-Hispanic white	1377 (80.04)	1282 (83.52)	95 (43.08)	
Non-Hispanic black	645 (7.97)	472 (6.42)	173 (24.42)	
Other	559 (8.63)	422 (7.48)	137 (20.83)	
Marital status, n (%)				< 0.001
Married	1569 (62.28)	1353 (64.11)	216 (42.88)	
Widowed/divorced	1025 (30.71)	807 (29.17)	218 (47.09)	
Never married	161 (4.35)	130 (4.04)	31 (7.56)	
Living with a partner	75 (2.66)	61 (2.68)	14 (2.48)	
Education, n (%)				< 0.001
Less than high school	715 (15.89)	408 (12.21)	307 (54.93)	
High school/GED	672 (22.45)	575 (22.27)	97 (24.35)	
College or above	1443 (61.66)	1368 (65.52)	75 (20.72)	
Annual family income, n (%)				< 0.001
< $20,000	755 (17.34)	530 (14.89)	225 (43.36)	
≥ $20,000	2075 (82.66)	1821 (85.11)	254 (56.64)	
Sleep disorders, n (%)	337 (11.86)	284 (11.94)	53 (10.96)	0.638
Sleep time, Mean (SE)	7.17 (0.02)	7.17 (0.03)	7.10 (0.09)	0.493
Smoking status, n (%)	1437 (50.34)	1180 (50.25)	257 (51.24)	0.787
Alcohol intake, n (%)	898 (27.27)	718 (25.86)	180 (42.24)	< 0.001
Work activity, n (%)				< 0.001
Vigorous	302 (12.72)	263 (13.35)	39 (6.01)	
Moderate	553 (21.81)	498 (22.80)	55 (11.31)	
Other	1975 (65.48)	1590 (63.85)	385 (82.68)	
Recreational activity, n (%)				< 0.001
Vigorous	258 (11.16)	246 (12.06)	12 (1.67)	
Moderate	917 (33.69)	802 (34.89)	115 (20.95)	
Other	1655 (55.15)	1303 (53.06)	352 (77.38)	
Depression, n (%)	258 (7.23)	175 (6.19)	83 (18.23)	< 0.001
Hypertension, n (%)	1766 (58.83)	1430 (57.38)	336 (74.18)	< 0.001
Diabetes, n (%)	705 (20.68)	530 (19.16)	175 (36.76)	< 0.001
Stroke, n (%)	199 (6.51)	143 (5.95)	56 (12.52)	< 0.001
Heart failure, n (%)	201 (6.70)	142 (5.76)	59 (16.70)	< 0.001
Coronary heart disease, n (%)	264 (9.62)	222 (9.56)	42 (10.20)	0.735
Heart attack, n (%)	242 (8.56)	199 (8.40)	43 (10.29)	0.298
MedDiet Score, Mean (SE)	4.60 (0.10)	4.64 (0.10)	4.21 (0.16)	0.003
WBC count, Mean (SE)	6.96 (0.07)	6.93 (0.06)	7.34 (0.18)	0.012
Lymphocyte count, Mean (SE)	1.91 (0.03)	1.90 (0.03)	2.05 (0.08)	0.043
Neutrophil count, Mean (SE)	4.22 (0.04)	4.20 (0.04)	4.44 (0.12)	0.052
NLR, Mean (SE)	2.51 (0.04)	2.50 (0.04)	2.55 (0.10)	0.653
PLR, Mean (SE)	131.25 (1.90)	131.72 (1.95)	126.28 (3.70)	0.144
NAR, Mean (SE)	1.01 (0.01)	1.00 (0.01)	1.09 (0.03)	0.007

*BMI* Body mass index, *GED* general education development, *WBC* White blood cell, *MedDiet* Mediterranean diet, *NLR* Neutrophil–lymphocyte ratio, *PLR* Platelet-lymphocyte ratio, *NAR* Neutrophil-albumin ratio

^a^Values were baseline values except when otherwise specified. Individual and global standardized age-dependent cognitive z-scores <  − 1 were characterized as “low cognitive performance” for their respective cognitive measure

We also found that 2351 (83.07%) people had normal cognitive performance, and 479 (16.93%) people had low cognitive performance. In the low cognitive performance group, age (*P* < 0.001), the proportions of non-married status (*P* < 0.001), below college education (*P* < 0.001), alcohol intake (*P* < 0.001), depression (*P* < 0.001), hypertension (*P* < 0.001), diabetes (*P* < 0.001), stroke (*P* = 0.003), and heart failure (*P* < 0.001), and the inflammatory markers of WBC count (*P* = 0.003), lymphocyte count (*P* = 0.003), and NAR (*P* = 0.003) were all significantly higher than the group with normal cognitive performance. Additionally, the proportion of annual family income ≥ $ 20,000 (*P* < 0.001) and the MedDiet score (*P* = 0.003) were significantly lower for people with low cognitive performance versus those of normal cognitive performance. The two groups exhibited significant differences in race (*P* < 0.001), work activity (*P* < 0.001), and recreational activity (*P* < 0.001) (Table 2).

### Association of the MedDiet adherence score and inflammatory markers with low cognitive performance

As presented in Table 3, Model 1 showed that the higher MedDiet adherence score correlated with the lower risk of low cognitive performance [odds ratio (OR) = 0.92, 95% confidence interval (CI): 0.87–0.97, *P* = 0.002). Conversely, increases in WBC count (OR = 1.17, 95% CI: 1.07–1.29, *P* < 0.001), neutrophil count (OR = 1.15, 95% CI: 1.01–1.30, *P* = 0.025) and NAR (OR = 1.21, 95% CI: 1.08–1.36, *P* < 0.001) were associated with the greater risk of low cognitive performance. The relations of low cognitive performance to the MedDiet adherence score, WBC count and NAR were unaltered in all models (all *P* < 0.05). The association between neutrophil count and low cognitive performance became non-significant in Model 2 (*P* = 0.073) and Model 3 (*P* = 0.070).

**Table 3:** Association of the MedDiet adherence score and inflammatory markers with low cognitive performance

Variables	Model 1		Model 2		Model 3	
	**OR (95% CI)**	***P***	**OR (95% CI)**	***P***	**OR (95% CI)**	***P***
**MedDiet adherence score**	0.92 (0.87–0.97)	0.002	0.92 (0.87–0.97)	0.002	0.891(0.831–0.955)	< 0.001
**Inflammatory marker**						
WBC count	1.17 (1.07–1.29)	< 0.001	1.15 (1.05–1.27)	0.002	1.16 (1.02–1.33)	0.017
Lymphocyte count	1.10 (0.99–1.23)	0.071	1.11 (0.96–1.27)	0.150	1.07 (0.97–1.17)	0.111
Neutrophil count	1.15 (1.01–1.30)	0.025	1.12 (0.99–1.27)	0.073	1.18 (0.98–1.43)	0.070
NLR	1.03 (0.91–1.17)	0.632	0.98 (0.85–1.13)	0.761	1.03 (0.89–1.18)	0.687
PLR	0.90 (0.76–1.06)	0.179	0.89 (0.75–1.04)	0.129	0.95 (0.82–1.10)	0.496
NAR	1.21 (1.08–1.36)	< 0.001	1.18 (1.05–1.32)	0.004	1.22 (1.02–1.45)	0.020

*MedDiet* Mediterranean diet, *WBC* White blood cell, *NLR* Neutrophil–lymphocyte ratio, *PLR* platelet-lymphocyte ratio, *NAR* Neutrophil-albumin ratio, *OR* Odds ratio, *CI* confidence interval

Model 1, not adjusted for covariates

Model 2, adjusted for age and gender

Model 3, adjusted for age, gender, BMI, race/Hispanic origin, marital status, education, annual family income, sleep disorders, alcohol intake, recreational activities, depression, hypertension, diabetes, stroke, and heart failure

### Optimal MedDiet adherence score threshold

**Fig. 2:**
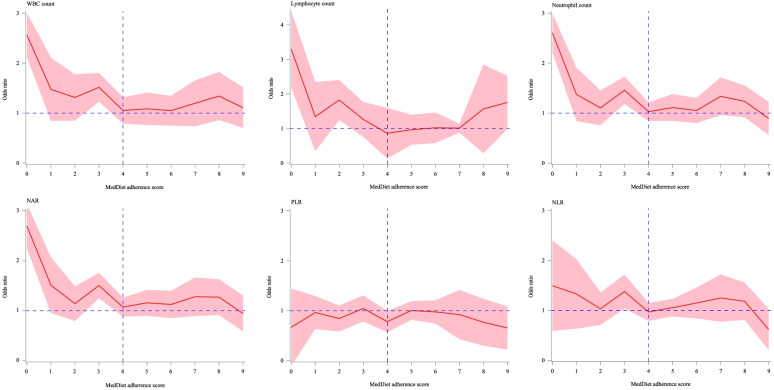
Association of inflammatory markers with low cognitive performance under different MedDiet adherence scores. MedDiet, Mediterranean diet; WBC, white blood cell; NLR, neutrophil–lymphocyte ratio; PLR, platelet-lymphocyte ratio; NAR, neutrophil-albumin ratio

By analyzing the associations of inflammatory markers with low cognitive performance at each MedDiet adherence score level, the results demonstrated that when the MedDiet adherence score raised to 4, normalized OR values for WBC count, lymphocyte count, neutrophil count, NLR, and NAR dropped significantly and approached 1 for the first time, so we chose 4 as the optimal MedDiet adherence score threshold. Then individuals with the MedDiet adherence score < 4 were classified into the low MedDiet adherence group, and those with the MedDiet adherence score ≥ 4 were classified into the high MedDiet adherence group (Fig. 2).

### Association of inflammatory markers with low cognitive performance in the high and low MedDiet adherence groups

As shown in Table 4, in the low MedDiet adherence group, higher WBC count (OR = 1.44, 95% CI: 1.09–1.90, *P* = 0.008), neutrophil count (OR = 1.30, 95% CI: 1.03–1.65, *P* = 0.023), and NAR (OR = 1.34, 95% CI: 1.06–1.70, *P* = 0.012) all correlated with a higher risk of low cognitive performance according to Model 3. In the high MedDiet adherence group, Model 3 illustrated that an elevation in PLR was linked to a reduced risk of low cognitive performance (OR = 0.86, 95% CI: 0.74–1.00, *P* = 0.036) (Table 4).

**Table 4:** Association between inflammatory markers and low cognitive performance in the high and low MedDiet adherence groups

Variables	Model 1		Model 2		Model 3	
	**OR (95% CI)**	***P***	**OR (95% CI)**	***P***	**OR (95% CI)**	***P***
**Low MedDiet adherence group**^**a**^						
WBC count	1.47 (1.11–1.95)	0.005	1.46 (1.11–1.92)	0.005	1.44 (1.09–1.90)	0.008
Lymphocyte count	1.37 (0.90–2.11)	0.132	1.48 (0.97–2.27)	0.059	1.45 (0.92–2.29)	0.093
Neutrophil count	1.32 (1.04–1.67)	0.016	1.30 (1.03–1.64)	0.020	1.30 (1.03–1.65)	0.023
NLR	1.21 (0.99–1.48)	0.050	1.17 (0.97–1.43)	0.096	1.16 (0.93–1.43)	0.166
PLR	0.92 (0.75–1.13)	0.382	0.92 (0.75–1.13)	0.402	0.91 (0.73–1.13)	0.372
NAR	1.36 (1.08–1.73)	0.007	1.35 (1.07–1.69)	0.008	1.34 (1.06–1.70)	0.012
**High MedDiet adherence group**						
WBC count	1.15 (0.98–1.35)	0.087	1.13 (0.96–1.32)	0.119	1.08 (0.93–1.25)	0.292
Lymphocyte count	1.08 (0.82–1.44)	0.568	1.17 (0.74–1.85)	0.485	1.17 (0.74–1.86)	0.485
Neutrophil count	1.12 (1.00–1.26)	0.044	1.09 (0.97–1.22)	0.132	1.05 (0.93–1.19)	0.411
NLR	1.05 (0.97–1.14)	0.215	0.97 (0.91–1.10)	0.940	0.97 (0.88–1.07)	0.533
PLR	0.87 (0.75–1.01)	0.051	0.86 (0.74–1.00)	0.042	0.86 (0.74–1.00)	0.036
NAR	1.16 (1.03–1.30)	0.009	1.13 (1.01–1.27)	0.033	1.08 (0.97–1.22)	0.159

*MedDiet* Mediterranean diet, *WBC* White blood cell, *NLR* Neutrophil–lymphocyte ratio, *PLR* Platelet-lymphocyte ratio, *NAR* Neutrophil-albumin ratio, *OR* Odds ratio, *CI* Confidence interval

^a^Individuals with the adherence score < 4 were classified into the low MedDiet adherence group, and individuals with the MedDiet adherence score ≥ 4 were classified into the high MedDiet adherence group

Model 1, not adjusted for covariates

Model 2, adjusted for age and gender

Model 3, adjusted for age, gender, BMI, race/Hispanic origin, marital status, education, annual family income, sleep disorders, alcohol intake, recreational activities, depression, hypertension, diabetes, stroke, and heart failure

**Fig. 3:**
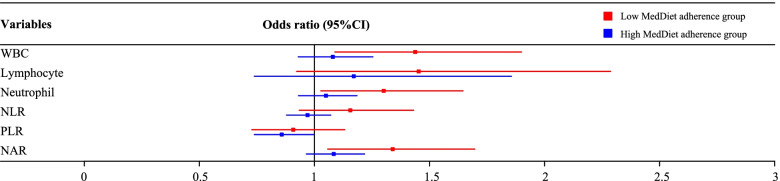
Risks of low cognitive performance in the high and low MedDiet adherence groups. MedDiet, Mediterranean diet; WBC, white blood cell; NLR, neutrophil–lymphocyte ratio; PLR, platelet-lymphocyte ratio; NAR, neutrophil-albumin ratio; CI, confidence interval. Individuals with the adherence score < 4 were classified into the low MedDiet adherence group, and individuals with the MedDiet adherence score ≥ 4 were classified into the high MedDiet adherence group

In addition, when each corresponding inflammatory marker increased equally, the risk of low cognitive performance in individuals with low MedDiet adherence was higher than that in those with high MedDiet adherence (Fig. 3). Based on the above findings from Table 4 and the significant differences found in the associations of WBC count, neutrophil count and NAR with low cognitive performance between the low and high MedDiet adherence groups (all *P* < 0.001) from Table 5, the negative association between inflammatory markers and cognitive performance was significantly weakened in older adults whose MedDiet adherence score ≥ 4. Further, subgroup analysis showed that this weakened negative association when the MedDiet adherence score ≥ 4 also existed among male, non-Hispanic white, normal-weight, overweight, moderate work activity, moderate recreational activity, non-depression, hypertension, non-hypertension, non-diabetes, non-stroke, non-heart failure, non-coronary heart disease, or non-heart attack subpopulations of older adults (Supplementary Tables 1, 2, 3, 4, 5, 6, 7, 8, 9, 10, 11 and 12).

**Table 5:** Difference in the association of inflammatory markers and low cognitive performance between the low and high MedDiet adherence groups

Variables	Low MedDiet adherence group^a^	High MedDiet adherence group	*P*
	**OR (95%CI)**	**OR (95%CI)**	
WBC count	1.44 (1.09–1.90)	1.08 (0.93–1.25)	< 0.001
Lymphocyte count	1.45 (0.92–2.29)	1.17 (0.74–1.86)	0.055
Neutrophil count	1.30 (1.03–1.65)	1.05 (0.93–1.19)	< 0.001
NLR	1.16 (0.93–1.43)	0.97 (0.88–1.07)	< 0.001
PLR	0.91 (0.73–1.13)	0.86 (0.74–1.00)	0.180
NAR	1.34 (1.06–1.70)	1.08 (0.97–1.22)	< 0.001

*MedDiet* Mediterranean diet, *WBC* White blood cell, *NLR* Neutrophil–lymphocyte ratio, *PLR* Platelet-lymphocyte ratio, *NAR* Neutrophil-albumin ratio, *OR* Odds ratio, *CI* Confidence interval

^a^Individuals with the adherence score < 4 were classified into the low MedDiet adherence group, and individuals with the MedDiet adherence score ≥ 4 were classified into the high MedDiet adherence group

## Discussion

The current study was the first to explore the optimal MedDiet adherence score threshold for the association between inflammatory markers and cognitive performance and used it to divide individuals into high and low MedDiet adherence populations. It was identified that 4 was the optimal threshold, and the association of inflammatory markers and low cognitive performance became weak in the high MedDiet adherence population (the adherence score ≥ 4). On the contrary, the association between them became strong in the low MedDiet adherence population (the adherence score < 4).

By using the optimal MedDiet adherence score threshold, we distinguished the high MedDiet adherence population from the low MedDiet adherence population and inferred that higher MedDiet adherence may benefit the cognitive performance of older adults by altering the association of inflammation markers with cognitive performance. This inference cohered with the views of many existing studies [25–37]. In addition, the finding that higher MedDiet adherence is associated with better cognitive function can not only be applied to the United States but also Sweden (a country located in Northern Europe) [37] and Greece (a country located in the Mediterranean) [25]. If Chinese seniors can adapt to the MedDiet or a diversified diet based on wheat with similar ingredients of the MedDiet, they may reduce the risk of cognitive decline [27]. The MedDiet is also effective in slowing the rate of cognitive decline in blacks [28]. However, some researchers suggested that MedDiet adherence had no relation to cognitive decline among the elderly [38, 39]. This may be caused by extensive methodological heterogeneity in the research design. For example, in the study of Crichton et al. [39], overall MedDiet adherence was shown to not correlate with cognitive function in individuals aged 40 to 65. Qin et al. [27] observed that a higher MedDiet adherence score was, only in participants aged 65 and above, significantly associated with a slower rate of cognitive decline. In our study, the average age of participants was older than the subjects described above. The age difference may more favorably reflect the relationship between the MedDiet and cognitive performance, as the MedDiet may have a greater impact on cognitive health later rather than earlier in life. In general, the use of different outcome measures, varied research samples, and measured target domains may affect the result of the association between the MedDiet and cognitive performance [33].

Furthermore, we found that the cognitive performance of older adults with low MedDiet adherence was significantly negatively related to some inflammatory indicators (WBC, Neutrophil, and NAR), and the relationship became weak in the high MedDiet adherence group. It means that the MedDiet may have a beneficial impact on cognition by anti-inflammatory effects. Many studies were in favor of this conjecture [17, 40, 41], because the protective effects of the MedDiet may be attributed to the high polyphenol concentration contained in wine and vegetables, which are known for anti-inflammatory capacity. This study also indicated that among male, non-Hispanic white, normal-weight, overweight, moderate work activity, moderate recreational activity, non-depression, hypertension, non-hypertension, non-diabetes, non-stroke, non-heart failure, non-coronary heart disease, or non-heart attack subpopulations of older adults, this weakened relationship between inflammatory indicators and cognitive performance when the MedDiet adherence score ≥ 4 still existed, suggesting that these subpopulations may protect their cognitive performance by enhancing MedDiet adherence with the MedDiet adherence score over 4.

Our study had several strengths. The finding of the optimal MedDiet adherence score threshold indicated that if the minimum level of MedDiet adherence for the elderly exceeds the optimal threshold, they may protect or maintain cognitive health at a lower cost and with less effort to change their eating habits. This may have significant implications for public health, such as modifying dietary recommendations for the elderly and effectively promoting the MedDiet in the world. In addition, the proposal of the optimal threshold for MedDiet adherence may also help researchers in studying MedDiet adherence-relevant issues to effectively distinguish between the high and low MedDiet adherence groups and to facilitate further studies.

A few limitations of the present study needed to be noted. First of all, due to the nature of the cross-sectional study, causal relationship could not be determined between MedDiet adherence, inflammation markers, and cognitive performance of the elderly, and we cannot rule out the possibility that low cognition has a potential influence on food decision, which may cause low MedDiet adherence. More studies are warranted to verify our findings and explore the cause and effect. Moreover, some inflammatory biomarkers which are related to cognitive function, such as CRP [42], pentraxin 3, and interleukin-2 [43], were not investigated in the NHANES database. These inflammatory biomarkers should be taken into consideration in future research.

## Conclusion

The optimal threshold for the MedDiet adherence score was 4, and the negative association between inflammation and cognitive performance could be weakened in older adults whose MedDiet adherence score was ≥ 4. More investigations are required to support our findings.

## Supplementary Information

**Additional file 1: Table S1.** Difference in the association of inflammatory markers and low cognitive performance between the low and high MedDiet adherence groups with different genders.

**Additional file 2: Table S2.** Difference in the association of inflammatory markers and low cognitive performance between the low and high MedDiet adherence groups with different races.

**Additional file 3: Table S3.** Difference in the association of inflammatory markers and low cognitive performance between the low and high MedDiet adherence groups with different BMIs.

**Additional file 4: Table S4.** Difference in the association of inflammatory markers and low cognitive performance between the low and high MedDiet adherence groups with different work activities.

**Additional file 5: Table S5.** Difference in the association of inflammatory markers and low cognitive performance between the low and high MedDiet adherence groups with different recreational activities.

**Additional file 6: Table S6.** Difference in the association of inflammatory markers and low cognitive performance between the low and high MedDiet adherence groups with/without depression.

**Additional file 7: Table S7.** Difference in the association of inflammatory markers and low cognitive performance between the low and high MedDiet adherence groups with/without hypertension.

**Additional file 8: Table S8.** Difference in the association of inflammatory markers and low cognitive performance between the low and high MedDiet adherence groups with/without diabetes.

**Additional file 9: Table S9.** Difference in the association of inflammatory markers and low cognitive performance between the low and high MedDiet adherence groups with/without stroke.

**Additional file 10: Table S10.** Difference in the association of inflammatory markers and low cognitive performance between the low and high MedDiet adherence groups with/without heart failure.

**Additional file 11: Table S11.** Difference in the association of inflammatory markers and low cognitive performance between the low and high MedDiet adherence groups with/without coronary heart disease.

**Additional file 12: Table S12.** Difference in the association of inflammatory markers and low cognitive performance between the low and high MedDiet adherence groups with/without heart attack.

## Data Availability

The datasets generated and/or analyzed during the current study are available in the NHANES database, https://www.cdc.gov/nchs/nhanes/.

## Abbreviations

MedDiet: Mediterranean diet
NHANES: National Health and Nutrition Examination Survey
FPED: Food Patterns Equivalents Database
CERAD: Consortium to Establish a Registry for Alzheimer’s Disease
AFT: Animal Fluency Test
DSST: Digit Symbol Substitution Test
