# Supplementary material for: MedDiet adherence score for the association between inflammatory markers and cognitive performance in the elderly: a study of the NHANES 2011–2014
Source: BMC Geriatr. 2022 Jun 21;22:511. doi: 10.1186/s12877-022-03140-1 (PMC9215079; doi:10.1186/s12877-022-03140-1)
Supplement: Supplementary file 1 — Additional file 1: Table S1. Difference in the association of inflammatory markers and low cognitive performance between the low and high MedDiet adherence groups with different genders. [file 12877_2022_3140_MOESM1_ESM.docx]

**Supplementary Table 1.** Difference in the association of inflammatory markers and low cognitive performance between the low and high MedDiet adherence groups with different genders

| **Groups** | **Variables** | **Low MedDiet adherence group^a^** | **High MedDiet adherence group** | ***P*** |
| --- | --- | --- | --- | --- |
|  |  | **OR (95%CI)** | **OR (95%CI)** |  |
| Male | WBC count | 1.58 (1.07-2.34) | 0.97 (0.85-1.11) | <0.001 |
|  | Lymphocyte count | 1.56 (0.81-3.01) | 1.06 (0.75-1.50) | <0.001 |
|  | Neutrophil count | 1.38 (0.97-1.95) | 0.95 (0.80-1.13) | <0.001 |
|  | NLR | 1.22 (0.90-1.66) | 0.94 (0.75-1.18) | <0.001 |
|  | PLR | 0.93 (0.70-1.22) | 0.85 (0.70-1.03) | 0.090 |
|  | NAR | 1.40 (1.00-1.96) | 0.99 (0.84-1.18) | <0.001 |
| Female | WBC count | 1.31 (0.92-1.86) | 1.25 (0.96-1.64) | <0.001 |
|  | Lymphocyte count | 1.33 (0.66-2.70) | 1.39 (0.87-2.21) | 0.662 |
|  | Neutrophil count | 1.23 (0.91-1.66) | 1.16 (0.93-1.44) | 0.206 |
|  | NLR | 1.09 (0.79-1.49) | 1.00 (0.86-1.16) | <0.001 |
|  | PLR | 0.89 (0.67-1.18) | 0.87 (0.71-1.08) | <0.001 |
|  | NAR | 1.28 (0.97-1.71) | 1.18 (0.95-1.48) | <0.001 |

MedDiet, Mediterranean diet; WBC, white blood cell; NLR, neutrophil-lymphocyte ratio; PLR, platelet-lymphocyte ratio; NAR, neutrophil-albumin ratio; OR, odds ratio; CI, confidence interval.

^a^ Individuals with the adherence score <4 were classified into the low MedDiet adherence group, and individuals with the MedDiet adherence score ≥4 were classified into the high MedDiet adherence group.
