# Supplementary material for: MedDiet adherence score for the association between inflammatory markers and cognitive performance in the elderly: a study of the NHANES 2011–2014
Source: BMC Geriatr. 2022 Jun 21;22:511. doi: 10.1186/s12877-022-03140-1 (PMC9215079; doi:10.1186/s12877-022-03140-1)
Supplement: Supplementary file 2 — Additional file 2: Table S2. Difference in the association of inflammatory markers and low cognitive performance between the low and high MedDiet adherence groups with different races. [file 12877_2022_3140_MOESM2_ESM.docx]

**Supplementary Table 2.** Difference in the association of inflammatory markers and low cognitive performance between the low and high MedDiet adherence groups with different races

| **Groups** | **Variables** | **Low MedDiet adherence group^a^** | **High MedDiet adherence group** | ***P*** |
| --- | --- | --- | --- | --- |
|  |  | **OR (95%CI)** | **OR (95%CI)** |  |
| Mexican American | WBC count | 2.57 (1.50-4.38) | 1.14 (0.66-1.97) | 0.003 |
|  | Lymphocyte count | 4.93 (2.52-9.65) | 1.46 (0.68-3.05) | 0.049 |
|  | Neutrophil count | 2.18 (1.27-3.75) | 1.05 (0.63-1.74) | <0.001 |
|  | NLR | 1.29 (0.39-4.24) | 1.16 (0.55-2.42) | 0.466 |
|  | PLR | 0.62 (0.35-1.09) | 1.00 (0.68-1.47) | <0.001 |
|  | NAR | 2.80 (1.65-4.76) | 1.12 (0.69-1.81) | 0.001 |
| Non-Hispanic white | WBC count | 1.56 (1.12-2.19) | 1.18 (0.95-1.46) | <0.001 |
|  | Lymphocyte count | 1.20 (0.75-1.91) | 1.06 (0.80-1.39) | 0.001 |
|  | Neutrophil count | 1.44 (1.07-1.94) | 1.18 (1.02-1.36) | <0.001 |
|  | NLR | 1.37 (1.06-1.76) | 1.04 (0.92-1.18) | <0.001 |
|  | PLR | 0.97 (0.75-1.26) | 0.88 (0.72-1.06) | 0.012 |
|  | NAR | 1.48 (1.10-1.99) | 1.20 (1.04-1.39) | 0.001 |
| Non-Hispanic black | WBC count | 1.29 (0.81-2.05) | 1.03 (0.77-1.38) | <0.001 |
|  | Lymphocyte count | 1.94 (0.76-4.90) | 1.20 (0.73-1.97) | 0.039 |
|  | Neutrophil count | 1.17 (0.77-1.78) | 1.01 (0.76-1.33) | <0.001 |
|  | NLR | 0.72 (0.31-1.67) | 1.06 (0.71-1.60) | <0.001 |
|  | PLR | 0.71 (0.47-1.09) | 1.09 (0.74-1.60) | <0.001 |
|  | NAR | 1.21 (0.76-1.94) | 1.06 (0.79-1.42) | 0.001 |
| Other | WBC count | 0.94 (0.51-1.75) | 0.87 (0.63-1.22) | 0.197 |
|  | Lymphocyte count | 2.58 (1.02-6.50) | 1.13 (0.63-2.02) | 0.016 |
|  | Neutrophil count | 0.75 (0.43-1.33) | 0.83 (0.62-1.12) | 0.252 |
|  | NLR | 0.32 (0.14-0.70) | 0.94 (0.72-1.24) | <0.001 |
|  | PLR | 0.61 (0.40-0.93) | 0.82 (0.62-1.09) | 0.006 |
|  | NAR | 0.80 (0.47-1.37) | 0.89 (0.68-1.17) | 0.073 |

MedDiet, Mediterranean diet; WBC, white blood cell; NLR, neutrophil-lymphocyte ratio; PLR, platelet-lymphocyte ratio; NAR, neutrophil-albumin ratio; OR, odds ratio; CI, confidence interval.

^a^ Individuals with the adherence score <4 were classified into the low MedDiet adherence group, and individuals with the MedDiet adherence score ≥4 were classified into the high MedDiet adherence group.
