# Supplementary material for: MedDiet adherence score for the association between inflammatory markers and cognitive performance in the elderly: a study of the NHANES 2011–2014
Source: BMC Geriatr. 2022 Jun 21;22:511. doi: 10.1186/s12877-022-03140-1 (PMC9215079; doi:10.1186/s12877-022-03140-1)
Supplement: Supplementary file 3 — Additional file 3: Table S3. Difference in the association of inflammatory markers and low cognitive performance between the low and high MedDiet adherence groups with different BMIs. [file 12877_2022_3140_MOESM3_ESM.docx]

**Supplementary Table 3.** Difference in the association of inflammatory markers and low cognitive performance between the low and high MedDiet adherence groups with different BMIs

| **Groups** | **Variables** | **Low MedDiet adherence group^a^** | **High MedDiet adherence group** | ***P*** |
| --- | --- | --- | --- | --- |
|  |  | **OR (95%CI)** | **OR (95%CI)** |  |
| Underweight | WBC count | 0.86 (0.86-0.86) | 0.604(0.078-4.697) | 0.500 |
|  | Lymphocyte count | 0.27 (0.274-0.274) | 0.00 (0.00-0.04) | 0.512 |
|  | Neutrophil count | 1.09 (1.090-1.090) | 1.16 (0.22-6.01) | 0.647 |
|  | NLR | - | 3.46 (1.16-10.34) | - |
|  | PLR | 0.87 (0.87-0.87) | 1.46 (0.98-2.19) | 0.002 |
|  | NAR | 1.63 (1.63-1.63) | 1.52 (0.33-7.11) | 0.855 |
| Normal-weight | WBC count | 2.01 (1.12-3.59) | 1.05 (0.76-1.45) | <0.001 |
|  | Lymphocyte count | 0.80 (0.42-1.53) | 0.94 (0.72-1.23) | 0.001 |
|  | Neutrophil count | 1.95 (1.20-3.17) | 1.05 (0.78-1.40) | <0.001 |
|  | NLR | 2.31 (1.28-4.16) | 1.09 (0.81-1.47) | <0.001 |
|  | PLR | 1.28 (0.90-1.83) | 1.04 (0.79-1.37) | <0.001 |
|  | NAR | 2.04 (1.28-3.24) | 1.07 (0.80-1.44) | <0.001 |
| Overweight | WBC count | 2.07 (1.26-3.41) | 1.18 (0.80-1.75) | 0.001 |
|  | Lymphocyte count | 3.40 (1.02-11.35) | 1.06 (0.73-1.54) | 0.004 |
|  | Neutrophil count | 1.48 (1.04-2.11) | 1.21 (0.95-1.55) | 0.022 |
|  | NLR | 0.88 (0.51-1.52) | 0.92 (0.78-1.08) | 0.141 |
|  | PLR | 0.74 (0.49-1.11) | 0.81 (0.63-1.04) | 0.271 |
|  | NAR | 1.48 (1.03-2.12) | 1.22 (0.93-1.59) | 0.033 |
| Obese | WBC count | 1.21 (0.74-1.99) | 1.31 (1.01-1.71) | 0.444 |
|  | Lymphocyte count | 1.04 (0.93-1.16) | 1.38 (0.79-2.41) | 0.092 |
|  | Neutrophil count | 1.20 (0.75-1.91) | 1.24 (1.01-1.51) | 0.665 |
|  | NLR | 1.29 (0.86-1.94) | 1.11 (0.89-1.39) | 0.004 |
|  | PLR | 1.10 (0.80-1.51) | 0.83 (0.66-1.05) | <0.001 |
|  | NAR | 1.25 (0.78-1.99) | 1.28 (1.04-1.59) | 0.758 |

MedDiet, Mediterranean diet; WBC, white blood cell; NLR, neutrophil-lymphocyte ratio; PLR, platelet-lymphocyte ratio; NAR, neutrophil-albumin ratio; OR, odds ratio; CI, confidence interval.

^a^ Individuals with the adherence score <4 were classified into the low MedDiet adherence group, and individuals with the MedDiet adherence score ≥4 were classified into the high MedDiet adherence group.
