# Supplementary material for: MedDiet adherence score for the association between inflammatory markers and cognitive performance in the elderly: a study of the NHANES 2011–2014
Source: BMC Geriatr. 2022 Jun 21;22:511. doi: 10.1186/s12877-022-03140-1 (PMC9215079; doi:10.1186/s12877-022-03140-1)
Supplement: Supplementary file 4 — Additional file 4: Table S4. Difference in the association of inflammatory markers and low cognitive performance between the low and high MedDiet adherence groups with different work activities. [file 12877_2022_3140_MOESM4_ESM.docx]

**Supplementary Table 4.** Difference in the association of inflammatory markers and low cognitive performance between the low and high MedDiet adherence groups with different work activities

| **Groups** | **Variables** | **Low MedDiet adherence group^a^** | **High MedDiet adherence group** | ***P*** |
| --- | --- | --- | --- | --- |
|  |  | **OR (95%CI)** | **OR (95%CI)** |  |
| Vigorous work activity | WBC count | 2.93(1.39-6.17) | 1.85 (0.96-3.55) | 0.358 |
|  | Lymphocyte count | 1.98 (0.59-6.69) | 0.57 (0.22-1.49) | 0.018 |
|  | Neutrophil count | 2.41 (1.21-4.82) | 2.59 (1.51-4.42) | 0.898 |
|  | NLR | 2.90 (0.66-12.67) | 2.87 (1.54-5.37) | 0.994 |
|  | PLR | 1.00 (0.57-1.70) | 1.19 (0.72-1.98) | 0.030 |
|  | NAR | 2.5 (1.18-5.43) | 3.19 (1.70-5.98) | 0.748 |
| Moderate work activity | WBC count | 2.06 (1.22-3.47) | 1.43 (0.97-2.11) | 0.101 |
|  | Lymphocyte count | 0.91 (0.40-2.06) | 1.79 (0.81-3.94) | 0.063 |
|  | Neutrophil count | 1.99 (1.30-3.06) | 1.24 (0.87-1.77) | 0.003 |
|  | NLR | 1.79 (1.19-2.71) | 1.06 (0.77-1.46) | <0.001 |
|  | PLR | 1.00 (0.74-1.35) | 0.89 (0.70-1.13) | 0.003 |
|  | NAR | 2.03 (1.35-3.04) | 1.19 (0.83-1.71) | <0.001 |
| Other work activity | WBC count | 1.35 (0.94-1.93) | 1.08 (0.89-1.31) | <0.001 |
|  | Lymphocyte count | 1.37 (0.84-2.22) | 1.11 (0.62-1.99) | 0.041 |
|  | Neutrophil count | 1.22 (0.91-1.64) | 1.03 (0.87-1.22) | <0.001 |
|  | NLR | 1.12 (0.83-1.50) | 0.94 (0.79-1.11) | <0.001 |
|  | PLR | 0.95 (0.69-1.32) | 0.87 (0.72-1.05) | 0.032 |
|  | NAR | 1.26 (0.94-1.70) | 1.07 (0.92-1.26) | <0.001 |

MedDiet, Mediterranean diet; WBC, white blood cell; NLR, neutrophil-lymphocyte ratio; PLR, platelet-lymphocyte ratio; NAR, neutrophil-albumin ratio; OR, odds ratio; CI, confidence interval.

^a^ Individuals with the adherence score <4 were classified into the low MedDiet adherence group, and individuals with the MedDiet adherence score ≥4 were classified into the high MedDiet adherence group.
