# Supplementary material for: MedDiet adherence score for the association between inflammatory markers and cognitive performance in the elderly: a study of the NHANES 2011–2014
Source: BMC Geriatr. 2022 Jun 21;22:511. doi: 10.1186/s12877-022-03140-1 (PMC9215079; doi:10.1186/s12877-022-03140-1)
Supplement: Supplementary file 5 — Additional file 5: Table S5. Difference in the association of inflammatory markers and low cognitive performance between the low and high MedDiet adherence groups with different recreational activities. [file 12877_2022_3140_MOESM5_ESM.docx]

**Supplementary Table 5.** Difference in the association of inflammatory markers and low cognitive performance between the low and high MedDiet adherence groups with different recreational activities

| Groups | **Variables** | **Low MedDiet adherence group^a^** | **High MedDiet adherence group** | ***P*** |
| --- | --- | --- | --- | --- |
|  |  | **OR (95%CI)** | **OR (95%CI)** |  |
| Vigorous recreational activity | WBC count | 1.59 (0.78-3.22) | 1.23 (0.71-2.12) | 0.154 |
|  | Lymphocyte count | 0.35 (0.10-1.25) | 3.86 (1.09-13.68) | 0.061 |
|  | Neutrophil count | 1.49 (0.81-2.75) | 0.91 (0.50-1.66) | <0.001 |
|  | NLR | 3.51 (1.17-10.47) | 0.51 (0.22-1.19) | 0.011 |
|  | PLR | 8.08 (2.37-27.62) | 0.34 (0.18-0.65) | 0.005 |
|  | NAR | 1.52 (0.80-2.87) | 0.93 (0.49-1.75) | 0.001 |
| Moderate recreational activity | WBC count | 1.60 (0.97-2.63) | 1.04 (0.78-1.40) | <0.001 |
|  | Lymphocyte count | 0.95 (0.53-1.70) | 1.07 (0.73-1.57) | <0.001 |
|  | Neutrophil count | 1.64 (1.05-2.55) | 1.03 (0.81-1.31) | <0.001 |
|  | NLR | 1.85 (1.01-3.42) | 1.07 (0.91-1.25) | <0.001 |
|  | PLR | 0.92 (0.67-1.28) | 1.07 (0.89-1.30) | <0.001 |
|  | NAR | 1.65 (1.06-2.57) | 1.07 (0.86-1.34) | <0.001 |
| Other recreational activity | WBC count | 1.41 (0.97-2.05) | 1.03 (0.88-1.21) | <0.001 |
|  | Lymphocyte count | 1.40 (0.79-2.48) | 1.01 (0.86-1.20) | <0.001 |
|  | Neutrophil count | 1.29 (0.96-1.75) | 1.03 (0.89-1.21) | <0.001 |
|  | NLR | 1.16 (0.92-1.45) | 0.95 (0.80-1.12) | <0.001 |
|  | PLR | 0.95 (0.72-1.24) | 0.82 (0.68-0.98) | 0.017 |
|  | NAR | 1.34 (1.00-1.79) | 1.05 (0.89-1.24) | <0.001 |

MedDiet, Mediterranean diet; WBC, white blood cell; NLR, neutrophil-lymphocyte ratio; PLR, platelet-lymphocyte ratio; NAR, neutrophil-albumin ratio; OR, odds ratio; CI, confidence interval.

^a^ Individuals with the adherence score <4 were classified into the low MedDiet adherence group, and individuals with the MedDiet adherence score ≥4 were classified into the high MedDiet adherence group.
