# Supplementary material for: MedDiet adherence score for the association between inflammatory markers and cognitive performance in the elderly: a study of the NHANES 2011–2014
Source: BMC Geriatr. 2022 Jun 21;22:511. doi: 10.1186/s12877-022-03140-1 (PMC9215079; doi:10.1186/s12877-022-03140-1)
Supplement: Supplementary file 6 — Additional file 6: Table S6. Difference in the association of inflammatory markers and low cognitive performance between the low and high MedDiet adherence groups with/without depression. [file 12877_2022_3140_MOESM6_ESM.docx]

**Supplementary Table 6.** Difference in the association of inflammatory markers and low cognitive performance between the low and high MedDiet adherence groups with/without [depression](C:/Program%20Files%20(x86)/Youdao/Dict/8.9.9.0/resultui/html/index.html#/javascript:;)

| **Groups** | **Variables** | **Low MedDiet adherence group^a^** | **High MedDiet adherence group** | ***P*** |
| --- | --- | --- | --- | --- |
|  |  | **OR (95%CI)** | **OR (95%CI)** |  |
| Depression | WBC count | 7.29 (2.36-22.53) | 1.62 (0.61-4.33) | 0.154 |
|  | Lymphocyte count | 16.01 (2.28-112.35) | 1.43 (0.49-4.11) | 0.209 |
|  | Neutrophil count | 3.19 (1.40-7.25) | 1.43 (0.63-3.26) | 0.117 |
|  | NLR | 1.02 (0.74-1.41) | 1.01 (0.55-1.85) | 0.017 |
|  | PLR | 0.64 (0.42-0.99) | 0.98 (0.65-1.49) | <0.001 |
|  | NAR | 2.64 (1.21-5.74) | 1.67 (0.70-4.00) | 0.366 |
| Non-[depression](C:/Program%20Files%20(x86)/Youdao/Dict/8.9.9.0/resultui/html/index.html#/javascript:;) | WBC count | 1.43 (1.07-1.90) | 1.11 (0.95-1.30) | <0.001 |
|  | Lymphocyte count | 1.18 (0.78-1.81) | 1.04 (0.86-1.25) | <0.001 |
|  | Neutrophil count | 1.34 (1.04-1.71) | 1.12 (0.98-1.27) | <0.001 |
|  | NLR | 1.28 (1.01-1.62) | 1.04 (0.93-1.17) | <0.001 |
|  | PLR | 0.95 (0.74-1.20) | 0.90 (0.77-1.06) | 0.121 |
|  | NAR | 1.38 (1.07-1.78) | 1.14 (1.00-1.29) | <0.001 |

MedDiet, Mediterranean diet; WBC, white blood cell; NLR, neutrophil-lymphocyte ratio; PLR, platelet-lymphocyte ratio; NAR, neutrophil-albumin ratio; OR, odds ratio; CI, confidence interval.

^a^ Individuals with the adherence score <4 were classified into the low MedDiet adherence group, and individuals with the MedDiet adherence score ≥4 were classified into the high MedDiet adherence group.
