# Supplementary material for: MedDiet adherence score for the association between inflammatory markers and cognitive performance in the elderly: a study of the NHANES 2011–2014
Source: BMC Geriatr. 2022 Jun 21;22:511. doi: 10.1186/s12877-022-03140-1 (PMC9215079; doi:10.1186/s12877-022-03140-1)
Supplement: Supplementary file 7 — Additional file 7: Table S7. Difference in the association of inflammatory markers and low cognitive performance between the low and high MedDiet adherence groups with/without hypertension. [file 12877_2022_3140_MOESM7_ESM.docx]

**Supplementary Table 7.** Difference in the association of inflammatory markers and low cognitive performance between the low and high MedDiet adherence groups with/without hypertension

| **Groups** | **Variables** | **Low MedDiet adherence group^a^** | **High MedDiet adherence group** | ***P*** |
| --- | --- | --- | --- | --- |
|  |  | **OR (95%CI)** | **OR (95%CI)** |  |
| Hypertension | WBC count | 1.50 (1.06-2.11) | 1.19 (0.99-1.45) | 0.002 |
|  | Lymphocyte count | 1.53 (0.83-2.82) | 1.30 (0.94-1.81) | 0.253 |
|  | Neutrophil count | 1.35 (1.01-1.80) | 1.15 (0.99-1.33) | 0.001 |
|  | NLR | 1.07 (0.81-1.42) | 1.01 (0.89-1.16) | <0.001 |
|  | PLR | 0.80 (0.64-1.00) | 0.89 (0.78-1.01) | 0.004 |
|  | NAR | 1.39 (1.05-1.84) | 1.17 (1.00-1.36) | 0.001 |
| Non-hypertension | WBC count | 1.49 (0.96-2.30) | 1.08 (0.80-1.46) | <0.001 |
|  | Lymphocyte count | 1.13(0.62-2.06) | 1.01 (0.84-1.21) | <0.001 |
|  | Neutrophil count | 1.41 (0.93-2.12) | 1.09 (0.83-1.44) | <0.001 |
|  | NLR | 1.82 (1.08-3.06) | 1.05 (0.83-1.33) | <0.001 |
|  | PLR | 1.24 (0.82-1.87) | 0.87 (0.66-1.16) | <0.001 |
|  | NAR | 1.46 (0.95-2.26) | 1.14 (0.86-1.49) | 0.001 |

MedDiet, Mediterranean diet; WBC, white blood cell; NLR, neutrophil-lymphocyte ratio; PLR, platelet-lymphocyte ratio; NAR, neutrophil-albumin ratio; OR, odds ratio; CI, confidence interval.

^a^ Individuals with the adherence score <4 were classified into the low MedDiet adherence group, and individuals with the MedDiet adherence score ≥4 were classified into the high MedDiet adherence group.
