# Supplementary material for: MedDiet adherence score for the association between inflammatory markers and cognitive performance in the elderly: a study of the NHANES 2011–2014
Source: BMC Geriatr. 2022 Jun 21;22:511. doi: 10.1186/s12877-022-03140-1 (PMC9215079; doi:10.1186/s12877-022-03140-1)
Supplement: Supplementary file 8 — Additional file 8: Table S8. Difference in the association of inflammatory markers and low cognitive performance between the low and high MedDiet adherence groups with/without diabetes. [file 12877_2022_3140_MOESM8_ESM.docx]

**Supplementary Table 8.** Difference in the association of inflammatory markers and low cognitive performance between the low and high MedDiet adherence groups with/without diabetes

| **Groups** | **Variables** | **Low MedDiet adherence group^a^** | **High MedDiet adherence group** | ***P*** |
| --- | --- | --- | --- | --- |
|  |  | **OR (95%CI)** | **OR (95%CI)** |  |
| Diabetes | WBC count | 0.91 (0.50-1.63) | 0.92 (0.65-1.29) | 0.771 |
|  | Lymphocyte count | 0.89 (0.37-2.12) | 1.17 (0.68-2.03) | 0.002 |
|  | Neutrophil count | 0.92 (0.601-1.407) | 0.94 (0.70-1.25) | 0.610 |
|  | NLR | 1.08 (0.776-1.499) | 0.97 (0.84-1.12) | <0.001 |
|  | PLR | 1.55 (0.945-2.553) | 0.88 (0.77-1.01) | <0.001 |
|  | NAR | 1.01 (0.643-1.576) | 0.97 (0.72-1.30) | 0.001 |
| Non-diabetes | WBC count | 1.66 (1.162-2.372) | 1.16 (0.91-1.47) | <0.001 |
|  | Lymphocyte count | 1.31 (0.820-2.100) | 1.06 (0.79-1.43) | <0.001 |
|  | Neutrophil count | 1.52 (1.085-2.125) | 1.14 (0.95-1.37) | <0.001 |
|  | NLR | 1.37 (0.977-1.927) | 1.01 (0.84-1.23) | <0.001 |
|  | PLR | 0.93 (0.742-1.158) | 0.90 (0.72-1.11) | 0.340 |
|  | NAR | 1.57 (1.111-2.211) | 1.16 (0.97-1.39) | <0.001 |

MedDiet, Mediterranean diet; WBC, white blood cell; NLR, neutrophil-lymphocyte ratio; PLR, platelet-lymphocyte ratio; NAR, neutrophil-albumin ratio; OR, odds ratio; CI, confidence interval.

^a^ Individuals with the adherence score <4 were classified into the low MedDiet adherence group, and individuals with the MedDiet adherence score ≥4 were classified into the high MedDiet adherence group.
