# Supplementary material for: MedDiet adherence score for the association between inflammatory markers and cognitive performance in the elderly: a study of the NHANES 2011–2014
Source: BMC Geriatr. 2022 Jun 21;22:511. doi: 10.1186/s12877-022-03140-1 (PMC9215079; doi:10.1186/s12877-022-03140-1)
Supplement: Supplementary file 9 — Additional file 9: Table S9. Difference in the association of inflammatory markers and low cognitive performance between the low and high MedDiet adherence groups with/without stroke. [file 12877_2022_3140_MOESM9_ESM.docx]

**Supplementary Table S9.** Difference in the association of inflammatory markers and low cognitive performance between the low and high MedDiet adherence groups with/without stroke.

| **Groups** | **Variables** | **Low MedDiet adherence group^a^** | **High MedDiet adherence group** | ***P*** |
| --- | --- | --- | --- | --- |
|  |  | **OR (95%CI)** | **OR (95%CI)** |  |
| Stroke | WBC count | 2.24 (1.22-4.11) | 1.25 (0.79-1.98) | 0.017 |
|  | Lymphocyte count | 7.52 (2.58-21.94) | 0.90 (0.33-2.46) | 0.047 |
|  | Neutrophil count | 1.26 (0.77-2.05) | 1.48 (0.96-2.28) | 0.402 |
|  | NLR | 1.00 (0.86-1.16) | 0.96 (0.75-1.23) | 0.007 |
|  | PLR | 0.80 (0.70-0.92) | 0.78 (0.55-1.11) | 0.817 |
|  | NAR | 1.42 (0.90-2.21) | 1.40 (0.96-2.05) | 0.952 |
| Non-stroke | WBC count | 1.46 (1.11-1.92) | 1.14 (0.94-1.39) | <0.001 |
|  | Lymphocyte count | 1.15 (0.79-1.68) | 1.07 (0.76-1.49) | 0.033 |
|  | Neutrophil count | 1.38 (1.08-1.76) | 1.12 (0.98-1.29) | <0.001 |
|  | NLR | 1.35 (1.09-1.68) | 1.04 (0.93-1.18) | <0.001 |
|  | PLR | 1.01 (0.77-1.33) | 0.91 (0.78-1.08) | <0.001 |
|  | NAR | 1.42 (1.11-1.82) | 1.15 (1.01-1.32) | <0.001 |

MedDiet, Mediterranean diet; WBC, white blood cell; NLR, neutrophil-lymphocyte ratio; PLR, platelet-lymphocyte ratio; NAR, neutrophil-albumin ratio; OR, odds ratio; CI, confidence interval.

^a^ Individuals with the adherence score <4 were classified into the low MedDiet adherence group, and individuals with the MedDiet adherence score ≥4 were classified into the high MedDiet adherence group.
