# Supplementary material for: MedDiet adherence score for the association between inflammatory markers and cognitive performance in the elderly: a study of the NHANES 2011–2014
Source: BMC Geriatr. 2022 Jun 21;22:511. doi: 10.1186/s12877-022-03140-1 (PMC9215079; doi:10.1186/s12877-022-03140-1)
Supplement: Supplementary file 10 — Additional file 10: Table S10. Difference in the association of inflammatory markers and low cognitive performance between the low and high MedDiet adherence groups with/without heart failure. [file 12877_2022_3140_MOESM10_ESM.docx]

**Supplementary Table S10.** Difference in the association of inflammatory markers and low cognitive performance between the low and high MedDiet adherence groups with/without heart failure.

| **Groups** | **Variables** | **Low MedDiet adherence group^a^** | **High MedDiet adherence group** | ***P*** |
| --- | --- | --- | --- | --- |
|  |  | **OR (95%CI)** | **OR (95%CI)** |  |
| Heart failure | WBC count | 8.57 (1.10-66.94) | 0.69 (0.35-1.36) | 0.038 |
|  | Lymphocyte count | 6.93 (0.11-458.60) | 0.62 (0.13-3.05) | 0.314 |
|  | Neutrophil count | 3.37 (1.12-10.15) | 0.83 (0.51-1.35) | 0.001 |
|  | NLR | 0.62 (0.25-1.54) | 1.00 (0.52-1.93) | 0.006 |
|  | PLR | 0.56 (0.24-1.30) | 1.13 (0.69-1.84) | <0.001 |
|  | NAR | 2.74 (1.04-7.17) | 0.89 (0.53-1.48) | 0.001 |
| Non-heart failure | WBC count | 1.42 (1.06-1.92) | 1.20 (0.99-1.46) | 0.014 |
|  | Lymphocyte count | 1.23 (0.79-1.93) | 1.32 (0.91-1.91) | 0.587 |
|  | Neutrophil count | 1.33 (1.03-1.71) | 1.13 (0.98-1.29) | <0.001 |
|  | NLR | 1.29 (1.02-1.63) | 1.03 (0.92-1.14) | <0.001 |
|  | PLR | 0.95 (0.75-1.20) | 0.87 (0.73-1.03) | 0.031 |
|  | NAR | 1.37 (1.06-1.78) | 1.15 (1.01-1.32) | <0.001 |

MedDiet, Mediterranean diet; WBC, white blood cell; NLR, neutrophil-lymphocyte ratio; PLR, platelet-lymphocyte ratio; NAR, neutrophil-albumin ratio; OR, odds ratio; CI, confidence interval.

^a^ Individuals with the adherence score <4 were classified into the low MedDiet adherence group, and individuals with the MedDiet adherence score ≥4 were classified into the high MedDiet adherence group.
