# Supplementary material for: MedDiet adherence score for the association between inflammatory markers and cognitive performance in the elderly: a study of the NHANES 2011–2014
Source: BMC Geriatr. 2022 Jun 21;22:511. doi: 10.1186/s12877-022-03140-1 (PMC9215079; doi:10.1186/s12877-022-03140-1)
Supplement: Supplementary file 11 — Additional file 11: Table S11. Difference in the association of inflammatory markers and low cognitive performance between the low and high MedDiet adherence groups with/without coronary heart disease. [file 12877_2022_3140_MOESM11_ESM.docx]

**Supplementary Table S11.** Difference in the association of inflammatory markers and low cognitive performance between the low and high MedDiet adherence groups with/without coronary heart disease.

| **Groups** | **Variables** | **Low MedDiet adherence group^a^** | **High MedDiet adherence group** | ***P*** |
| --- | --- | --- | --- | --- |
|  |  | **OR (95%CI)** | **OR (95%CI)** |  |
| Coronary heart disease | WBC count | 1.46 (0.79-2.68) | 1.24 (0.84-1.84) | 0.239 |
|  | Lymphocyte count | 0.84 (0.22-3.19) | 1.36 (0.70-2.63) | 0.015 |
|  | Neutrophil count | 1.45 (0.98-2.16) | 1.08 (0.81-1.45) | <0.001 |
|  | NLR | 1.17 (0.86-1.60) | 0.99 (0.76-1.28) | <0.001 |
|  | PLR | 1.42 (0.75-2.70) | 0.81 (0.58-1.13) | <0.001 |
|  | NAR | 1.59 (1.03-2.45) | 1.09 (0.81-1.48) | <0.001 |
| Non-coronary heart disease | WBC count | 1.53 (1.11-2.09) | 1.12 (0.92-1.37) | <0.001 |
|  | Lymphocyte count | 1.29 (0.83-2.01) | 1.05 (0.85-1.30) | <0.001 |
|  | Neutrophil count | 1.40 (1.07-1.85) | 1.13 (0.97-1.31) | <0.001 |
|  | NLR | 1.31 (1.01-1.71) | 1.04 (0.91-1.18) | <0.001 |
|  | PLR | 0.94 (0.74-1.19) | 0.91 (0.78-1.07) | 0.295 |
|  | NAR | 1.45 (1.09-1.92) | 1.16 (1.00-1.34) | <0.001 |

MedDiet, Mediterranean diet; WBC, white blood cell; NLR, neutrophil-lymphocyte ratio; PLR, platelet-lymphocyte ratio; NAR, neutrophil-albumin ratio; OR, odds ratio; CI, confidence interval.

^a^ Individuals with the adherence score <4 were classified into the low MedDiet adherence group, and individuals with the MedDiet adherence score ≥4 were classified into the high MedDiet adherence group.
