# Supplementary material for: MedDiet adherence score for the association between inflammatory markers and cognitive performance in the elderly: a study of the NHANES 2011–2014
Source: BMC Geriatr. 2022 Jun 21;22:511. doi: 10.1186/s12877-022-03140-1 (PMC9215079; doi:10.1186/s12877-022-03140-1)
Supplement: Supplementary file 12 — Additional file 12: Table S12. Difference in the association of inflammatory markers and low cognitive performance between the low and high MedDiet adherence groups with/without heart attack. [file 12877_2022_3140_MOESM12_ESM.docx]

**Supplementary Table 12.** Difference in the association of inflammatory markers and low cognitive performance between the low and high MedDiet adherence groups with/without heart attack

| **Groups** | **Variables** | **Low MedDiet adherence group^a^** | **High MedDiet adherence group** | ***P*** |
| --- | --- | --- | --- | --- |
|  |  | **OR (95%CI)** | **OR (95%CI)** |  |
| Heart attack | WBC count | 0.58 (0.24-1.43) | 0.93 (0.55-1.57) | 0.010 |
|  | Lymphocyte count | 0.36 (0.09-1.44) | 1.13 (0.42-3.02) | 0.045 |
|  | Neutrophil count | 0.70 (0.37-1.32) | 0.90 (0.59-1.37) | 0.010 |
|  | NLR | 0.93 (0.61-1.41) | 0.99 (0.73-1.35) | <0.001 |
|  | PLR | 1.70 (0.64-4.50) | 1.05 (0.77-1.42) | <0.001 |
|  | NAR | 0.77 (0.39-1.49) | 0.88 (0.57-1.36) | 0.122 |
| Non-heart attack | WBC count | 1.59 (1.16-2.19) | 1.16 (0.94-1.43) | <0.001 |
|  | Lymphocyte count | 1.36 (0.86-2.16) | 1.07 (0.79-1.45) | <0.001 |
|  | Neutrophil count | 1.46 (1.11-1.92) | 1.15 (0.99-1.34) | <0.001 |
|  | NLR | 1.32 (1.02-1.71) | 1.04 (0.90-1.19) | <0.001 |
|  | PLR | 0.93 (0.73-1.18) | 0.87 (0.74-1.02) | 0.112 |
|  | NAR | 1.50 (1.14-1.98) | 1.19 (1.02-1.38) | <0.001 |

MedDiet, Mediterranean diet; WBC, white blood cell; NLR, neutrophil-lymphocyte ratio; PLR, platelet-lymphocyte ratio; NAR, neutrophil-albumin ratio; OR, odds ratio; CI, confidence interval.

^a^ Individuals with the adherence score <4 were classified into the low MedDiet adherence group, and individuals with the MedDiet adherence score ≥4 were classified into the high MedDiet adherence group.
